# Associations between disease activity, social support and health-related quality of life in patients with inflammatory bowel diseases: the mediating role of psychological symptoms

**DOI:** 10.1186/s12876-020-1166-y

**Published:** 2020-01-14

**Authors:** Hanlin Fu, Atipatsa Chiwanda Kaminga, Yan Peng, Tiejian Feng, Tingting Wang, Xiaobing Wu, Tubao Yang

**Affiliations:** 1 Department of Epidemiology and Health Statistics, XiangYa School of Public Health, Central South University, NO. 238 Shangmayuanling Road, Kaifu District, Changsha, 410078 Hunan Province China; 2 Department of Dermatology and Venereal Disease, Shenzhen Center for Chronic Disease Control, Province518020, Shenzhen, Guangdong China; 3 Department of Mathematics and Statistics, Mzuzu University, Private Bag 201, Luwinga, Mzuzu 2, Malawi; 4 Department of Gastroenterology, The Third Xiangya Hospital, Central South University, Changsha, 410013 Hunan Province China

**Keywords:** Inflammatory bowel disease, Health-related quality of life, Disease activity, Psychological symptoms, Social support

## Abstract

**Background:**

Previous studies have indicated that disease activity, psychological symptoms and social support were associated with health-related quality of life (HRQoL) in patients with inflammatory bowel diseases(IBD). However, it is unclear how disease activity, psychological symptoms and social support interact to affect HRQoL. The main purpose of this study was to examine the mediation effect of psychological symptoms in the relationship between disease activity, social support and HRQoL.

**Methods:**

This was a cross-sectional study, which collected data using convenience sampling, between December 2016 and March 2018, from the Third XiangyaHospital of Central South University in Changsha, China. An online self-administered questionnaire (including demographic and clinical information), Inflammatory Bowel Disease Questionnaire, Disease Activity Indices scale, Hospital Anxiety and Depression Scale and Social Support Rating Scale, were administered to each participant. Descriptive statistics and Pearson’s correlations were used to summarize data, whereas PROCESS analysis was performed to examine the pre-specified mediation effect.

**Results:**

A total of 199 patients with IBD were included. Disease activity indices (DAI) and hospital anxiety and depression (HAD) were negatively correlated with HRQoL (*β* = − 3.37, − 2.54 respectively, *P* < 0.001), while social support was positively correlated with HRQoL (*β* = 1.38, *P* < 0.01). HAD partially mediated the negative relationship between DAI and HRQoL (*β* = − 0.83, *P* < 0.001) with the mediation effect ratio of 24.6%, and completely mediated the positive relationship between social support and HRQoL (*β* = 1.20, *P* < 0.001).

**Conclusions:**

Psychological symptoms acted as a mediator in the relationship between disease activity, social support and HRQoL. Interventions to improve HRQoL in patients with IBD should take into account the mediation effect of psychological symptoms.

## Background

Inflammatory bowel diseases (IBD), including ulcerative colitis (UC) and Crohn’s disease (CD), are a group of chronic, non-specific inflammatory disorders of the gastrointestinal tract, whose etiology and pathogenesis have not been fully understood. The incidence and prevalence of IBD is increasing worldwide implying that there is need for more interventions to prevent and control this burden on people’s quality of life [1–3]. For example, with the development of industrialization, the twentieth century has witnessed a surging incidence and prevalence of IBD in Western countries [1]. Based on a recent systematic review, the incidence of IBD in the Western countries is up to 25 per 100,000 person-years, and the prevalence is up to 0.5%, which are the highest globally [2]. Moreover, the newly industrialized countries in Asia, the Middle East, and South America also experienced steep increases in the incidence and prevalence of IBD in the past decades [1, 3]. According to some prediction, IBD patients in China will reach 1.5 million by 2025.

IBD is characterized by no cure, low mortality and irregular alternating course of remission and relapse, which commonly occur between ages 15 and 40 and can persist throughout the patient’s lifetime [4]. The most common clinical symptoms of IBD are abdominal pain, diarrhea and bloody stool, usually complicated with extraintestinal manifestations. Due to lifelong medical treatment and loss of productivity, IBD causes a considerable financial burden on patients. It is estimated that the annual per-patient indirect costs of UC and CD are $1159.09~$14,135.64and $926.49~$6583.17, respectively [5]. In addition, IBD is associated with the presence of psychological disorders [6–8]. The physical, economical and psychosocial burden adversely affect patients’ personal health experience and daily life, leading to impaired health-related quality of life (HRQoL).

HRQoL is a broad multidimensional evaluation that incorporates both objective and subjective aspects, focusing on physical, psychosocial and role functioning, as well as mental health and general health perceptions [9, 10]. During recent years, a growing number of studies related to HRQoL in IBD have been published [11]. As with other chronic diseases, IBD patients suffered from poorer HRQoL relative to the general population. The main therapeutic goal for IBD was to control disease flares, alleviate symptoms and ultimately improve patient’s HRQoL. In clinical research and clinical practice, evaluation of HRQoL is favored and has been increasingly used by researchers and clinicians, in that it can provide an insight into patients’ perception of their health and treatment effectiveness, and also can be applied to compare effects of different treatment strategies [12]. Previous literatures have demonstrated that the HRQoL of IBD patients was affected by disease-related factors, psychosocial factors and demographic factors, with the last aspect somewhat controversial [13–15]. Based on the variables involved in the impairment of HRQoL of IBD patients, tailored interventions for improving HRQoL could be effectively developed.

Disease activity in IBD has been proved to have a confirmed effect on HRQoL [9, 11, 14, 16–18]. A large body of evidence consistently supported that patients with active IBD in relapse have significantly decreased HRQoL in contrast with patients in remission [14, 17–19]. Improvement of HRQoL can be achieved by diminishing disease activity or inducing remission. However, there was still a large number of IBD patients reporting low HRQoL despite being in remission [9, 20], signifying a possibility of other underlying determinants.

Psychological symptoms have been considered as other important determinants of HRQoL [11, 16, 19, 21–23]. For example, depression and anxiety were highly prevalent in patients with IBD, especially during relapse [11, 22, 23]. A systematic review and meta-analysis of 171 articles revealed that the prevalence of anxiety and depression symptoms among IBD patients was 35.1 and 21.6%, respectively [8]. In addition, the prevalence of depression symptoms was even higher (49%) among 2,325,226 IBD patients [6]. It has been suggested that IBD was susceptible to trigger or intensify an underlying psychiatric condition, which in turn contributed to the disease progression and flare [22]. Similar to other chronic disease, there was a strong evidence indicating that psychological distress was associated with a reduced HRQoL in IBD patients [16, 19, 21–23].

Social support, referred to as the support from family, friends and others, is identified as a protective factors for increased HRQoL across a variety of chronic diseases [24]. It was found that high levels of social support could help minimize pain and stress, provide a buffer against psychological distress, and enhance self-management [24]. Empirical studies have shown that IBD patients with lower perceived support had an impairment of HRQoL [13, 24]. However, literatures on the relationships between social support and IBD-related HRQoL were relatively lacking.

**Fig. 1:**
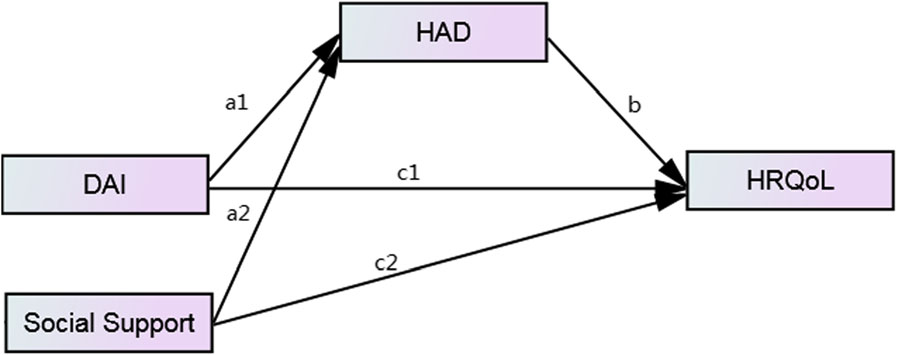
The theoretic mediation model relating DAI, Social Support, HAD and HRQoL. Abbreviations: DAI = disease activity indices, HAD = hospital anxiety and depression, HRQoL = health related quality of life

Since HRQoL could be affected by disease activity, psychological symptoms and social support, and psychological symptoms were associated with disease activity and social support, we conjectured that psychological symptoms may act as a mediator in the relationships between disease activity, social support and HRQoL. However, there was no research clarifying the mediation effect of psychological symptoms that may exist in IBD patients. Therefore, the current study aimed to (1) confirm the positive relationship between social support and HRQoL; (2) examine the mediating role of psychological symptoms in the relationships between disease activity, social support and HRQoL in IBD patients. Figure 1 presents the conceptual model.

## Methods

### Study design and participants

This was a cross-sectional study, which collected data using convenience sampling, between December 2016 and March 2018, at the Third XiangyaHospital of Central South University in Changsha, China.. Potential participants were those who came to the hospital wards of the department of gastroenterology. Inclusion criteria were: (1) diagnosed with IBD definitely, (2) aged≥18 years, (3) able to communicate and comprehend in relation to the questionnaires, and (4) volunteered to participate in the study. Exclusion criteria were: (1) refused to participate, (2) inability to comprehend or complete the questionnaires, (3) presence of severe co-existing diseases potentially affecting HRQoL, and (4) complicated with other gastrointestinal diseases. Informed consent was obtained from every participant and all questionnaires were self-administered online through mobilephones. In addition, participant who came to the department many times were investigated only once.

### Measures

#### Demographic and clinical information

A self-designed questionnaire was used to collect participants’ demographic and clinical data including gender, age, ethnicity, education, marital status, monthly income, place of residence, employment (study) status, physical exercise, type of IBD and disease course.

#### Health related quality of life

The Inflammatory Bowel Disease Questionnaire (IBDQ) is the most widely used disease-specific instrument designed and recommended to assess HRQoL in patients with IBD [11, 25]. It was initially developed by Guyatt et al. in 1989 [26], and now has been translated into and cross-culturally adapted in several different language versions. In each case, it has been shown to have good reliability and validity in many different regions and nations [19, 27–31]. The questionnaire consists of 32 items that divided into four domains: bowel symptoms (10 questions), systematic symptoms (5 questions), emotional function (12 questions) and social function (5 questions). Each question is rated on a 7-point Likert scale where 1 and 7 correspond to the worst and best function level, respectively. The total IBDQ score ranged from 32 to 224, with the higher score indicating a better HRQoL.

In the current study, the Chinese version of IBDQ, validated by Ren et al. [32], was used to measure the HRQoL of the subjects. Every subject was asked about their feeling over the past 2 weeks. The Chinese version of IBDQ has also been proved to be valid and reliable, with the Cronbach’s alpha of 0.95 in UC and 0.94 in CD, and the test–retest reliability ranged between 0.69–0.93.

#### Disease activity indices

The Walmsley simple clinical colitis activity index (SCCAI) [33] was used to assess the disease activity indices among patients with UC, whereas the Harvey–Bradshaw simple index (HBI) [34] was used to assess the same among patients with CD. These two scales are widely used for their good validity, reliability and convenience of operation. The SCCAI is calculated based on six parameters: stool frequency during the day and night, bloody stool, defecation urgency, general well-being and extraintestinal manifestations. Its total score ranges from 0~ > 16. The HBI is scored according to the general well-being, abdominal pain, frequency of diarrhea, abdominal mass (diagnosed by clinicians) and complications. The total score for HBI ranges from 0~ > 12. Disease activity for both SCCAI and HBI are classified as: remission- a score less than 4, and active-a score at least 4.

#### Psychological status

Psychological status was measured by the Chinese version of the Hospital Anxiety and Depression Scale (HADS). The HADS is specially designed for screening non-psychiatric patients. It has 14 items, of which 7 are developed for depression diagnosis, and the rest are for anxiety diagnosis. Each item is scored on a 4-point Likert scale ranging from 0 to 3. The total score for both anxiety and depression subscales range between 0 and 21. Using the HADS, depression and anxiety can be categorized into four levels as follows: normal (0–7), mild (8–10), moderate (11–15), and severe (16–21). The total score of HAD is calculated by summing the scores of all items from the two subscales. A patient with the total score ≥ 13 is considered to be pathological. The Cronbach’s alpha of the HADS is 0.933 and 0.870 for each subscale.

#### Social support

The Social Support Rating Scale (SSRS) developed by Xiao [35] was used to assess social support. The SSRS is a three-dimensional measurement with ten items that measures objective social support, subjective social support and support utilization. The scoring rules of each item is as follows: items 1–4 and 8–10 are scored from 1 to 4; item 5 is divided into 5 aspects and each aspect is also scored from 1 to 4, corresponding to “no” to “full support”, respectively, and the score of item 5 is the sum of the 5 aspects; items 6 and 7 are scored as 0 if the answer is “no source”, otherwise scored as the number of sources. The final score ranges from 12 to 64, with the higher score indicating a higher level of social support. The test–retest reliability of the SSRS is 0.92.

#### Statistical analysis

Data analyses were conducted using the Statistical Package for the Social Sciences (SPSS) version 23. Descriptive statistics were expressed as means and standard deviations (SD) for quantitative variables, and counts and percentages for categorical variables. A correlation matrix, performed by Pearson’s correlation analysis, was presented to determine the relationships among variables including DAI, social support, anxiety, depression, HAD and HRQoL. Two separate mediation analyses, using the PROCESS module 4 in SPSS macro [36], were conducted to examine whether the relationships between social support, DAI and HRQoL were mediated by HAD (Fig. 1). For both mediation analyses, HRQoL was used as the dependent variable, while social support and DAI were used as independent variables. The mediation effect of HAD were assessed while employing bootstrapping with 5000 samples. Direct, indirect and total effects with corresponding bias corrected bootstrap confidence intervals were finally provided. All tests were two-tailed, with statistical significance defined as *P* < 0.05.

## Results

### Descriptive statistics

A total of 199 patients with IBD were examined using the study tools. As shown in Table 1, subjects consisted of 114 (57.3%) males and 85 (42.7%) females. The mean age was 35.3 years (SD: 10.6), with about two in five between 25 and 35 years and the rest about equally split in other age groups. Majority of the subjects were Han nationality (80.9%) and married (69.8%). About one half of them had college or above education background (53.8%), had a monthly income of ¥2000~5000 (49.7%), lived in rural areas (54.3%), and did physical exercises at least once per week (47.2%). At the time of the survey, 44.2% were on sick leave or unemployed, 37.2% were in a state of work/study and sick leave alternate, and only 18.6% chose to continue work or study. As to disease characteristics, 56.8% were suffering from CD and 43.2% were suffering from UC. In addition, 61.3% of them had a disease course of 1~5 years, 23.1% < 1 year and 15.6% ≥6 years.

**Table 1:** Demographic and clinical characteristics of IBD patients (*N* = 199)

Variables	n	%
Gender		
male	114	57.3
female	85	42.7
Age, years (mean = 35.3 SD = 10.6)		
18~	29	14.6
25~	77	38.7
35~	42	21.1
45~	51	25.6
Ethnicity		
Han	161	80.9
Others	38	19.1
Education		
Primary school or below	17	8.5
Junior high school	45	22.6
Senior high school	30	15.1
College or above	107	53.8
Marital status		
Unmarried	46	23.1
Married	139	69.8
Divorced or widowed	14	7.0
Monthly income,		
≤ ¥1999	10	5.0
¥2000~	99	49.7
¥5000~	67	33.7
≥ ¥10,000	23	11.6
Place of residence		
City/County/Town	108	54.3
Rural	91	45.7
Employment (study) status		
Continue work or study	37	18.6
Sick leave/Unemployed	88	44.2
Work/study and sick leave alternate	74	37.2
Physical exercise		
Inactivity	26	13.1
< 4 times per month	41	20.6
≥ 1 times per week	94	47.2
≥ 1 times per day	38	19.1
Type of IBD		
UC	86	43.2
CD	113	56.8
Disease course, years		
< 1	46	23.1
1~	122	61.3
≥ 6	31	15.6
DAI		
**< 4**	28	14.1
≥ 4	171	85.9
Depression		
Normal	90	45.2
Mild	42	21.1
Moderate	56	28.1
Severe	11	5.5
Anxiety		
Normal	113	56.8
Mild	28	14.1
Moderate	51	25.6
Severe	7	3.5

*Abbreviations: IBD* Inflammatory bowel disease, *DAI* Disease activity indices, *SD* Standard deviation

The mean total score was 15.5 (SD: 9.0) for HADS, 7.4(SD: 4.6) for anxiety subscale, and 8.1(SD: 4.7) for depression subscale. With regard to anxiety and depression levels, 56.8 and 45.2% were identified as normal, 14.1 and 21.1% were mild, 25.6% and 28.1were moderate, and 3.5 and 5.5% were severe, respectively (Table 2).

**Table 2:** Correlations between involved variables(*r*^*a*^)

Variables	Mean(SD)	1	2	3	4	5	6
1.DAI	8.1(4.4)	1.000					
2.Social support	37.6(6.5)	–	1.000				
3.Anxiety	7.4(4.6)	0.544^*^	−0.407^*^	1.000			
4.Depression	8.1(4.7)	0.595^*^	−0.409^*^	0.914^*^	1.000		
5.HAD	15.5(9.0)	0.583^*^	−0.417^*^	–	–	1.000	
6. HRQoL	141.8(37.1)	−0.688^*^	0.338^*^	−0.792^*^	−0.814^*^	− 0.821^*^	1.000

^a^The Pearson’s correlation analysis were applied and the correlation coefficients were shown in the table

*P < 0.01*Abbreviations: DAI* Disease activity indices, *HAD* Hospital anxiety and depression, *HRQoL* Health related quality of life, *SD* Standard deviation

The mean score for DAI was 8.1 (SD: 4.4). Also, 171 participants (85.9%) scored 4 or above on DAI, indicating that their disease was in “active”. On the other hand, 28 participants (14.1%) scored less than 4, indicating that their disease was in “remission”. (Table 2).

In terms of social support and HRQoL, the mean scores of SSRS and IBDQ were 37.6 (SD: 6.5) and 141.8 (SD: 37.1), respectively. (Table 2).

### Correlations between variables

The correlations among DAI, social support, anxiety, depression, HAD and HRQoL were presented in Table 2. Anxiety symptoms, depression symptoms and HAD were negatively correlated with social support and HRQoL (*r* = − 0.792, *r* = − 0.814, and *r* = − 0.821, respectively, *P* < 0.01). In addition, they were significantly positively correlated with DAI (*r* = 0.544, *r* = 0.595, and *r* = 0.583, respectively, *P* < 0.01). Also, DAI was significantly negatively correlated with HRQoL (*r* = − 0.688, *P* < 0.01). Conversely, social support was significantly positively correlated with HRQoL(*r* = 0.338, *P* < 0.01).

### HAD as a mediator between DAI, social support and HRQoL

Two mediation models were examined to determine the mediation effects of HAD between DAI, social support and HRQoL. The results of each model were shown in Table 3.

**Table 3:** Mediation effect of DAI-HAD-HRQoL, and Social Support-HAD-HRQoL by PROCESS

Effect	Coeff.	t/Z	*P*	LLCI	ULCL
Mediation analysis 1: DAI-HAD-HRQoL					
Total effect	−5.54	−13.21	0.000^**^	−6.37	−4.71
Direct effect	−2.74	−7.14	0.000^**^	−3.49	−1.98
Indirect effect	−2.81	−7.81	0.000^**^	−3.63	−2.14
a1	1.10	9.97	0.000^**^	0.89	1.32
b	−2.54	−12.62	0.000^**^	−2.94	−2.15
Mediation analysis 2: Social Support-HAD-HRQoL					
Total effect	1.38	4.90	0.000^**^	0.82	1.93
Direct effect	0.18	0.79	0.428	−0.27	0.64
Indirect effect	1.20	5.65	0.000^**^	0.81	1.68
a2	−0.47	−6.34	0.000^**^	−0.62	− 0.32
b	−2.54	−12.62	0.000^**^	−2.94	−2.15

^**^P < 0.01*Abbreviations: DAI* Disease activity indices, *HAD* Hospital anxiety and depression, *HRQoL* Health related quality of life, *LLCI* Lower level confidence interval, *ULCL* Upper level confidence interval

In the model DAI-HAD-HRQoL, the total effect and direct effect of DAI on HRQoL were both significant (*β* = − 5.54 and *β* = − 2.74, respectively, *P* < 0.001). DAI was positively associated with HAD (*β* = 1.10, *P* < 0.001), while HAD was negatively related to HRQoL (*β* = − 2.54, *P* < 0.001), leading to the result that HAD partially mediated the negative relationship between DAI and HRQoL (*β* = − 2.81, *P* < 0.001). The mediation effect ratio was − 2.81/− 5.54 ≈ 50.7%.

**Fig. 2:**
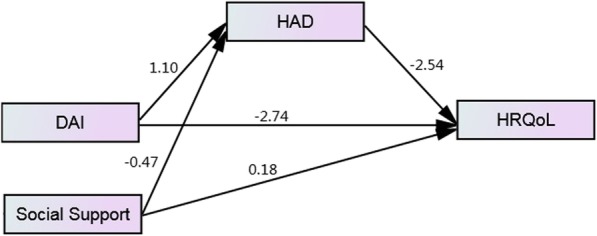
The mediation effect of HAD in the relation between DAI, Social Support and HRQoL. Abbreviations: DAI = disease activity indices, HAD = hospital anxiety and depression, HRQoL = health related quality of life

In the model Social Support-HAD-HRQoL, the total effect of social support on HRQoL was also significant (*β* = 1.38, *P* < 0.001). However, no significant direct effect was found between the two variables (*β* = 0.18, *P* = 0.428). Social support could negatively affect HAD (*β* = − 0.47, *P* < 0.001). The indirect effect of social support on HRQoL through HAD was 1.20 (*P* < 0.001). Therefore, HAD was a complete mediator between social support and HRQoL. Figure 2 shows the mediation model with the regression coefficients.

## Discussion

The purpose of the current study was to explore if social support was positively associated with HRQoL and if HAD mediates the relationships between DAI, social support and HRQoL in IBD patients. To the best of our knowledge, this is the first attempt to analyze the mediating role of HAD in the foregoing relationships, which could help better understand the mechanisms of the effect of DAI and social support on HRQoL, and assess the effectiveness of mediation interventions on expected outcomes. There were several points worth noting. First, HAD had a negative effect on HRQoL, which was in accordance with most previous studies [11, 16, 19, 21–23]. Psychological symptoms were common in IBD patients, and more than 40% of the participants reported different degrees of depression or anxiety symptoms, which is similar to results of other related domestic studies [37, 38]. It was suggested that the impact of psychological symptoms on impaired HRQoL was independent of other variables [19]. In addition, psychological symptoms likely contributed to poor treatment compliance, which further worsened outcomes for IBD patients and their HRQoL [6, 23]. In a systematic review and meta-analysis of 14 randomized controlled trials (RCT), psychological therapies were considered to have beneficial effects on HRQoL in IBD patients [7]. Therefore, it was important to routinely screen psychological symptoms early and provide effective interventions in order to promote HRQoL among this population.

Second, consistent with the existing literatures, our findings confirmed that disease activity was negatively associated with HRQoL [14, 17–19], which meant that the higher the DAI, the worse the HRQoL. Additionally, the current study suggested that disease activity not only directly affected the HRQoL of IBD patients, but also indirectly affected their HRQoL through the mediator, psychological symptoms, with the mediation effect ratio of 50.7%. The direct effect of DAI on HRQoL among IBD patients could be explained by the affected bowel functioning, somatic discomfort and increased pain experience [39]. As regards indirect effect, it was found that DAI positively contributed to HAD in the model, possibly owing to the fact that the patients with active IBD had to bear frequent physician visits, hospitalizations, side effects of treatment and so on [39], all of which caused a great burden on their mental health. Consequently, HRQoL was adversely affected through these indirect pathways. Nevertheless, reducing disease activity remains a priority for increasing IBD-related HRQoL.

Third, the current study confirmed that social support was positively related to HRQoL among the IBD patients, and this relationship was completely mediated by HAD. However, this is inconsistent with results of previous studies in other fields which both suggested a direct and indirect link between social support and HRQoL [40, 41]. It was inferred that the lack of the significant direct effect was likely confounded by other variables (e.g. gender, age, ethnicity, marital status, monthly income etc.), which has been validated but not presented in our study. Based on the Stress-Buffering Model, social support had an indirect effect on increased HRQoL through buffering the negative influence of psychological symptoms [42]. Therefore, enhancing social support which can be provided by family member, friends, coworkers and health professions should be valued as an important source to alleviate psychological symptoms and thereby improve HRQoL in IBD patients.

There are several limitations in the present study. First, the smaller sample size of this study was selected from one hospital only using convenience (non-probability) sampling method. Therefore, the reliability and generalizability of the results may be limited by selection bias. Therefore, more studies with recommended large sample size from multiple centers are required to ascertain the findings of this study. Second, due to the nature of the cross-sectional study, causal links among social support, DAI, HAD, and HRQoL could not be determined. The models, DAI-HAD-HRQoL and Social Support-HAD-HRQoL, were formed according to research experience and existing theories, however, other models, such as HAD-DAI-HRQoL and HAD-Social Support-HRQoL, cannot be ruled out. Longitudinal studies or randomized controlled trials (RCT) would be necessary to address this issue in the future. Third, the reliability and validity of the Chinese versions of HADS and SSRS used in this study was not evaluated for the studied population. However, both scales have been extensively used and validated in a variety of domestic studies and population, which has demonstrated good stability over time..

## Conclusion

Despite these limitations, this study has shown a strong evidence that social support was positively associated with HRQoL, while HAD and DAI were negatively associated with HRQoL in IBD patients. In addition, the mediating role of HAD in the relationships between DAI, social support and HRQoL was confirmed. The findings enhanced our understanding how DAI, social support and HAD may together influence HRQoL, and this was considerable for emphasizing the importance of psychological interventions in the improvement of HRQoL among IBD patients. Given the increasing incidence and prevalence of IBD and related impaired HRQoL, the findings of this study have practical implications on the development of interventions for promoting the HRQoL in patients with IBD.

## Data Availability

The datasets used and/or analysed during the current study are available from the corresponding author on reasonable request.
